# Oncogenic miR-20b-5p contributes to malignant behaviors of breast cancer stem cells by bidirectionally regulating CCND1 and E2F1

**DOI:** 10.1186/s12885-020-07395-y

**Published:** 2020-10-02

**Authors:** Liqin Xia, Feng Li, Jun Qiu, Zhongming Feng, Zihan Xu, Zhengtang Chen, Jianguo Sun

**Affiliations:** 1 Institute of Cancer, Xinqiao Hospital, Army Medical University, Chongqing, 400037 China; 2 West China-Guang’An Hospital, Sichuan University, Guang’an, 638001 Sichuan China; 3 Xiamen Humanity Hospital Fujian Medical University, Xiamen, 361000 Fujian China; 4 Chongqing Huamei Plastic Surgery Hosptial, Chongqing, 400037 China

**Keywords:** Breast cancer, miR-20b-5p, CCND1, E2F1, Cancer stem cells, Bidirectional regulation

## Abstract

**Background:**

Breast cancer is the leading cause of cancer mortality in women worldwide. Therefore, it is of great significance to identify the biological mechanism of tumorigenesis and explore the development of breast cancer to achieve a better prognosis for individuals suffering from breast cancer. MicroRNAs (miRNAs) have become a hot topic in cancer research, but the underlying mechanism of its involvement in cancer remains unclear.

**Methods:**

The miRNA profile between breast cancer stem cells (BCSCs, CD44^+^CD24^−/low^) and control MCF-7 breast cancer cells was obtained in a previous study. Based on biological analysis, miR-20b-5p was hypothesized to be a key factor due to the malignant behavior of BCSCs. Then, agomir-20b-5p and antagomir-20b-5p were transfected into MCF-7 and T47D breast cancer cells to detect cell migration, wound healing and proliferation, and lentivirus vectors silencing or overexpressing miR-20b-5p were transfected into T47D-CSCs to detect proliferation and apoptosis. The effect of miR-20b-5p on xenograft growth was investigated in vivo by transfection of a lentivirus-overexpression vector into T47D cells. The target genes were predicted by the online programs picTar, miRanda and TargetScan and verified by dual luciferase assay, and changes in protein expression were detected by western blot.

**Results:**

MiR-20b-5p had the highest degree in both the miRNA-gene network and miRNA-GO network to regulate BCSCs. Overexpression of miR-20b-5p significantly promoted the migration and wound healing ability of MCF-7 cells and T47D cells compared with the control (*P* < 0.05). In addition, miR-20b-5p facilitated the proliferation of MCF-7 cells and T47D-CSCs (*P* < 0.05) and inhibited the apoptosis of T47D-CSCs (*P* < 0.05). Moreover, miR-20b-5p promoted xenograft growth compared with the control group (*P* < 0.05). Accordingly, potential targets of both CCND1 and E2F1 were predicted by bioinformatics analysis. MiR-20b-5p directly targeted both CCND1 and E2F1 in a dual luciferase assay, while antagomir-20b-5p downregulated the protein levels of CCND1 and E2F1.

**Conclusions:**

Oncogenic miR-20b-5p was confirmed to promote the malignant behaviors of breast cancer cells and BCSCs. The underlying mechanism lies in that miR-20b-5p overall enhanced both CCND1 and E2F1 targets via bidirectional regulation probably involving direct downregulation and indirect upregulation.

## Background

Breast cancer is one of the most common cancers in women worldwide [1]. In recent years, although significant progress has been made in the diagnosis and treatment of malignant tumors, breast cancer remains the leading cause of cancer-related deaths in women, largely due to its high rate of relapse, metastasis, and drug resistance [2–5]. Therefore, it is of great significance to explore the mechanism of tumorigenesis in breast cancer and further identify key regulatory factors. Recent studies, including our previous findings, have demonstrated that miRNAs play an important role in regulating biological functions [6–8]. Because of their unique expression profile and special functions, miRNAs have been used as molecular markers to define the direction and processes of cell differentiation by regulating one or several target genes. MiRNAs can also control the self-renewal or proliferation of stem cells [9]. Abnormal changes in miRNAs have been discovered in breast cancer, and miRNAs have been demonstrated to be associated with the tumorigenesis and progression of breast cancer [8, 10]. Previous studies have confirmed that miRNAs play an important role in gene regulation in CD44^+^CD24^−/low^ breast cancer stem cells (BCSCs). For example, we found that miR-200C was significantly downregulated in BCSCs and played a critical role in their biological features [8]. In addition, let-7a plays an important role in BCSCs’ self-renewal by inhibiting the expression of H-Ras [11, 12].

In our previous research, we performed miRNA profiling between sorted CD44^+^CD24^−/low^ BCSCs and the control MCF-7 breast cancer cells [8]. Finally, miR-20b-5p was chosen for further study due to its highest degree of regulation. We found that miR-20b-5p belongs to the miR-106a-363 cluster, which together with the miR-17-92 cluster and miR-106b-25 cluster forms a large family of highly similar miRNAs called the miR-17 family [13]. In many human malignancies, members of the miR-17 family have been reported to accumulate in tumor cells and are speculated to exert oncogenic effects [14, 15], and miR-20b serves as a potential oncogene in gastric cancer [16], breast cancer [17, 18] and hepatocellular carcinoma [19]. However, there has been no studies exploring the regulatory role of miR-20b in BCSCs, and the underlying mechanism of miR-20b-5p remains unclear. This study aimed to investigate the role of the miR-20b-5p, a subtype of miR-20b family, in the regulation of the malignant behavior of BCSCs and to identify its target genes.

## Methods

### Induction culture of BCSCs

MCF-7 and T47D cells were cultured in DMEM-H (HyClone, USA) containing 10% fetal bovine serum (FBS) at 37 °C in a 5% CO_2_ incubator. Cells in the logarithmic growth phase, approximately 70–80% confluent, were harvested and digested into a single cell suspension by trypsin digestion.

The complete MammoCult™ medium containing 5% MammoCult™ proliferation supplement, 4 μg/mL heparin, and 0.48 μg/mL hydrocortisone was used. MCF-7 cells were resuspended in complete MammoCult™ medium, and T47D cells resuspended in complete DMEM/F-12 medium. The cell suspension (4 × 10^3^) was seeded into a 6-well plate and cultured at 37 °C and 5% CO_2_. After 7 days of culture, the spheres were collected and centrifuged at 350 g for 5 min, and the supernatant was discarded. Then, 1 mL of Accutase cell dispersion solution was added to digest the spheres, followed by adding 9 mL of sterile PBS solution and centrifugation at 350 g for 5 min. Finally, the supernatant was discarded, and the cells were collected.

CD44^+^CD24^−/low^ BCSCs were isolated from MCF-7 and T47D cells by staining with CD44-APC, CD24-PE and ESA-FITC (BD Pharmingen, USA) antibodies via FACS as described in our previous research [8, 20].

### miRNA profile and miRNA network

Both human miRNA microarray fabrication and hybridization were performed as described previously [8]. The miRNA profiles of both BCSCs and control MCF-7 breast cancer cells were obtained from CapitalBio Corporation (Beijing, China). All microarray data were uploaded and submitted to the public repository Gene Expression Omnibus (GEO) database (http://www.ncbi.nlm.nih.gov/geo/query/acc.cgi? acc = GSE68271). The differential miRNA profiles between BCSCs and MCF-7 cells were obtained [20].

To identify the miRNAs that regulate BCSCs, we established a miRNA-gene network and miRNA-GO network through analysis of the significant target genes and Gene Ontology (GO) terms performed by Shanghai Qiming Corporation (Shanghai, China).

### Target prediction by bioinformatics

Chromosome localization, sequence analysis and target prediction of the miRNAs were carried out by the online programs picTar (http://pictar.mdc-berlin.de/), miRanda (http://microrna.sanger.ac.uk), and TargetScan (http://www.targetscan.org). The mRNAs associated with cell proliferation and the cell cycle predicted by at least three algorithms were selected as putative targets. Then, the binding free energy (△G) of the hybridization between miRNAs and their 3′ UTR complementary sites was analyzed with the aid of Mfold software. The mRNAs able to bind with lower free energy at both 5′-70 bp and 3′-70 bp than their average random free energy were deemed to be accessible to specific miRNAs [20, 21].

### RNA isolation and quantitative real-time PCR (qRT-PCR)

Total RNA was extracted using TRIzol Reagent (Invitrogen, USA) according to the manufacturer’s instructions. After the concentration and purity of total RNA were determined, reverse transcription was performed using a PrimeScript RT reagent Kit (TaKaRa, Dalian, China). For qPCR analysis, cDNA was amplified with a SYBR Premix Ex Taq (TaKaRa) kit by using an AB 7500 Real-time PCR system. The relative gene expression was calculated by the 2^−ΔΔCt^ method. For miR-20b-5p qRT-PCR, the primers for miR-20b-5p (Forward: 5′-CAAAGTGCTCATAGTGCAG GTAG-3′, Reverse 5′-GCAAAGTGCTCATAGTGCAGG-3′) and U6 (Forward 5′-CTCGCTTCGGCAGCACA-3′, Reverse 5′-AACGCTTCACGAATTTGCGT-3′) were used. For qRT-PCR of potential target genes, primers are listed in Table 1.

**Table 1:** Primers of potential target genes

Gene	Forward primer	Reverse primer
CCND1	5′-CCCGCACGATTTCATTGAAC-3′	5′-GGCGGATTGGAAATGAACTTC-3′
E2F1	5′-ACCTCTTCGACTGTGACTTTG-3′	5′-GAGCATCTCTGGAAACCCTG-3′
MAPK1	5′-AGCGTATCAGCATGCCAC-3′	5′-GACCTCGAAGACGTTTCTCC-3′
STAT3	5′-GCTTCCCTGATTGTGACTG-3’	5′-CTGACAGATGTTGGAGATCACC-3’
R2b23b	5′-CTGACAGAGCAGTGGAGTATC-3’	5′-TGTTGCTGTCGTAGTTGCTG-3’
RAB5BR	5′-GCCAGTCCTAGCATCGTTATTG-3’	5′-TCACGTTCATAGCTGTCTTGG-3’
RABEP1	5′-GCCACAGTCTCTGAGAACACCAAG-3’	5′-GGAACTGGTGCTCATAGTCACGAA-3’
TAOK3	5′-CAAGAGACACGGAATGGACC-3’	5′-TCACGGACATGCTTGGAATG-3’
PPARRDR	5′-GCTTCCACTACGGTGTTCATG-3’	5′-CTTCTCGTACTCCAGCTTCATG-3’
XIAP	5′-AAACACCATCACTAACTAGAAGAATTG-3’	5′-CAAGTGATTTATAGTTGCTCCCAG-3’

### Transwell migration assay

Transient transfection of agomir-20b-5p and antagomir-20b-5p in MCF-7 and T47D breast cancer cells was performed as follows. After digestion, breast cancer cells were mixed with 1 mL DMEM-H containing 10% FBS and subjected to resuspension followed by cell counting. Then, the 24-well plates were inoculated at 4 × 10^4^ cells per well, containing 500 μL of medium, and placed in a 37 °C, 5% CO_2_ incubator overnight. The medium was removed the next day, and the cells were washed with PBS three times, and then 500 μL of Opti-MEM medium was added to each well. Next, Lipofectamine 2000 (Invitrogen, USA) was mixed with 1 μL of agomir of miR-20b-5p (agomir-20b-5p), antagomir of miR-20b-5p (antagomir-20b-5p) (Dharmacon, USA), or miR-control at a final concentration of 30 nM, and the cells were incubated for 24–48 h to achieve successful transfection.

The transwell chamber was placed in a 24-well plate. A total of 800 μL of DMEM-H containing 15% FBS was added to the lower chamber, and 200 μL transfected MCF-7 or T47D breast cancer cells (4 × 10^4^ cells resuspended in 3% DMEM-H) was added to the upper chamber and cultured in a 37 °C, 5% CO_2_ incubator for 24 h. After removal of the medium, the cells were fixed with 1 mL of 95% ethanol solution for 10 min, washed 3 times with 1 mL of PBS for 5 min each, and then stained with 1% crystal violet dye solution for 10 min. The crystal violet dye in the upper chamber was washed off under a small stream of water and gently wiped with a cotton swab. Finally, the cells were observed under an inverted microscope followed by imaging and counting (Leica, Germany). The test was repeated three times.

### Wound healing assay

Transient transfection of agomir-20b-5p and antagomir-20b-5p into MCF-7 and T47D breast cancer cells was performed as described above. First, 3–4 straight lines were drawn vertically on the back of the 6-well plate. Then, the cells were seeded according to the grouping and cultured in DMEM-H containing 10% FBS. When the transfected MCF-7 and T47D breast cancer cells reached 60–70% confluence, mitomycin was added for 2 h (final concentration was 10 ng/mL). After washing 3 times with PBS, the cells were scratched gently with a 100 μL pipette tip according to the marked lines, and then the detached cells were removed by washing with PBS. Under an inverted microscope (Leica, Germany), the imaging position was recorded at the 24 h and 48 h time points. The test was repeated three times.

### Proliferation assay in MCF-7 cells and T47D-CSCs

Transient transfection of agomir-20b-5p and antagomir-20b-5p into MCF-7 breast cancer cells was performed as described above. MCF-7 cells were incubated with 30 μM EdU, and cell proliferation was evaluated by flow cytometry using an EdU assay kit (GeneCopoeia, USA) according to the manufacturer’s instructions. The test was repeated five times.

Stable transfection of a lentivirus overexpression system was used in the T47D-CSC proliferation assays. The lentiviral vector (GV369) overexpressing miR-20b-5p was purchased from GeneChem (China). The miR-20b-5p overexpression vector was labeled T47D-CSCs/LV-miR-20b-5p, and the control vector was labeled T47D-CSCs/LV-NC. The induced T47D-CSCs were suspended in EpiCult-B serum-free medium, and Lipofectamine 2000 (Invitrogen, USA) was added together with T47D-CSCs/LV-miR-20b-5p or T47D-CSCs/LV-NC. Then, the viruses were harvested. Cultured cells were infected with 5 μg/mL polybrene and lentivirus (MOI = 50) for 24 h, and then the cells were incubated with fresh medium for another 48 h to establish stable cell lines. The proliferation assay was conducted as described above. The test was repeated five times.

Stable transfection of a lentivirus-miR-sponge construct was used in T47D-CSC proliferation assays. The miR-20b-5p shRNA sponge lentivirus for inhibition of miR-20b-5p was purchased from Hanbio Biotechnology (China). The tandem antisense sequence of miR-20b-5p (CTACCTGCACTATGAGCACTTTG) was synthesized and cloned into the shRNA lentiviral vector pHBLV-U6-mCherry-Puro (Hanbio Biotechnology, China). Then, the miR-20b-5p sponge construct and the empty vector were packaged and labeled T47D-CSCs/miR-20b-5p-sp and T47D-CSCs/vector-sp, respectively. The miR-20b-5p sponge shRNA lentiviral vector (Hanbio Biotechnology, China) or control lentiviral vector was incubated at a final concentration of 30 nM for 24–48 h. The proliferation assay in induced T47D-CSCs was conducted as described above. The test was repeated five times.

### Apoptosis assay in T47D-CSCs

The stable transfection of lentivirus overexpression and lentivirus-miR-sponge constructs was used to detect apoptosis in T47D-CSCs as described above. The induced T47D-CSCs were stained with anti-annexin V/7-AAD antibodies in binding buffer for 15 min and then analyzed for apoptosis by flow cytometry. The test was repeated five times.

### Animal experiments

The stable transfection of T47D cells overexpressing lentivirus was conducted as described above, and the cells were used in animal experiments. The vector overexpressing miR-20b-5p was labeled as T47D/LV-miR-20b-5p, and the control vector was labeled as T47D/LV-NC. All animal experiments were approved by the Institutional Animal Care and Use Committee of Xinqiao Hospital, Army Medical University. A total of 15 nude mice (4–6 weeks old) from the SPF Laboratory Animal Center of Xinqiao Hospital were randomly divided into the following three groups with 5 mice each: T47D control, T47D/LV-NC and T47D/LV-miR-20b-5p. A total of 1 × 10^7^ cells/100 μL of PBS were subcutaneously injected to establish a xenograft model. The tumor size was measured every 3 days and calculated according to the formula V = (length × width^2^)/2. After 4 weeks, the mice were sacrificed by cutting off the head after injection of Chloral hydrate, and the tumor tissues were collected for miR-20b-5p detection by qRT-PCR. All protocols involving mice were conducted in accordance with the animal care guidelines of Xinqiao Hospital.

### Dual luciferase assay

Two hundred ninety-three T cells were cultured with DMEM (supplemented with 10% FBS) to a cell density of 70–80%. We followed the protocol of our previous work for experimental design [22]. With Renilla luciferase as the internal reference, the RLU value determined for firefly luciferase was divided by the Renilla luciferase value. The vectors (GV306) for CCND1 and E2F1 were purchased from GeneChem (China). The inserted sequences were as follows: wild type CCND1 (CCND1-WT) and mutated CCND1 (CCND1-MT), wild type E2F1 (E2F1-WT) and mutated E2F1 (E2F1-MT) (Supplementary Fig. 1). A random sequence and miR-20b-5p mimics were used for negative control (NC) and miRNA overexpression, respectively. The experiment was divided into 4 groups: NC + CCND1-WT, MIMICS+CCND1-WT, NC + CCND1-MT, and MIMICS+CCND1-MT. The same experiment was performed with E2F1 reagents.

### Western blot analysis of target genes

Transfected cells were washed twice with PBS, lysed in RIPA buffer (Beyotime, China) containing a protease inhibitor cocktail (Roche, Switzerland) for 30 min and then centrifuged at 13000×g for 10 min. After boiling in loading buffer for 10 min, the supernatants were loaded onto SDS-PAGE gels and then transferred onto PVDF membranes (Millipore, USA). After blocking, the membranes were incubated separately with specific primary antibodies overnight at 4 °C, followed by incubation with goat anti-rabbit IgG or goat anti-mouse IgG antibodies (ZSGB-Bio, China) for 1 h at room temperature. Finally, immunoreactive proteins were visualized using a chemiluminescence detection system (FluorChem HD2, USA).

### Statistical analysis

Data were analyzed by one way ANOVA or t-test, and statistical analyses were performed using SPSS 21.0 and GraphPad Prism 5.0 software. The comparisons with a *P* < 0.05 were considered statistically significant.

## Results

### Key miRNAs screened by miRNA networks

**Fig. 1:**
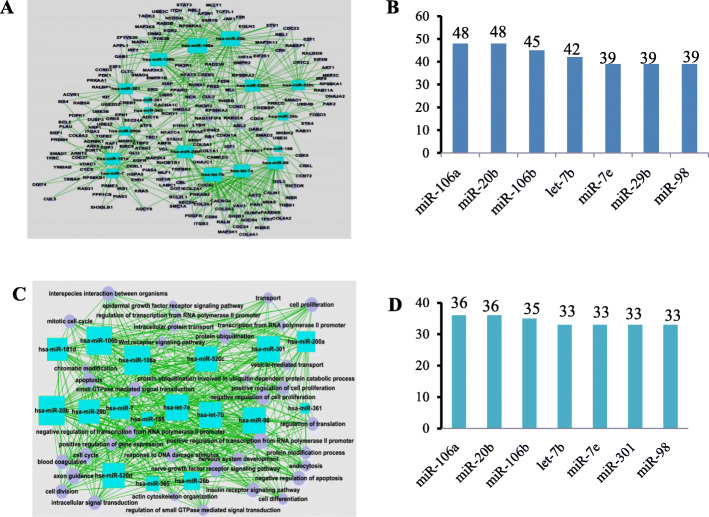
Screening of breast cancer stem cells (BCSCs)-associated miRNAs via miRNA networks. **a** In the miRNA-gene network, the rectangle in the figure represents upregulated miRNAs, the circle represents genes, and the line represents the regulatory relationship between miRNAs and genes; **b** The regulatory degrees of miRNAs in the miRNA-gene network; **c** In the miRNA-GO network, the rectangle in the figure represents miRNAs, the circle represents GO terms, and the straight line represents the regulatory relationship between miRNAs and GO terms. The more miRNAs regulating a GO term, the larger its area; **d** The regulatory degrees of miRNAs in the miRNAs-GO network

From the bioinformatics analysis on the miRNA profiles of BCSCs and counterpart of control breast cancer cells, we obtained two networks. In the miRNA-gene network (Fig. 1a), the crosstalk degrees of miR-106a-5p, miR-20b-5p, miR-106b-3p, let-7b-5p, miR-7e-5p, miR-29b-3p, and miR-98-5p ranked at the top, with an average degree of 42.86. Among them, the degrees of miR-106a-5p and miR-20b-5p reached 48 (Fig. 1b). In the miRNA-GO network (Fig. 1c), the crosstalk degrees of miR-106a-5p, miR-20b-5p, miR-106b-3p, let-7b-5p, miR-7e-5p, miR-29b-3p and miR-98-5p ranked at the top, with an average degree of 34.14. Among them, the degrees of miR-106a-5p and miR-20b-5p reached 36 (Fig. 1d). Based on the two networks above, it was amazing that both miR-106a-5p and miR-20b-5p had the same highest regulatory degrees, which was recorded and described in our previous work [20, 23]. Coincidentally, they are both located on the X chromosome (Xq26.2, GRCh37) and less than 1 kb apart. Moreover, miR-106a-5p and miR-20b-5p together with other molecules, such as miR-92a-2 and miR-363, constitute a cluster of miRNAs [24]. Our previous publication demonstrated that miR-106a-5p acted as a tumor suppressor gene and significantly inhibited the invasion and migration of breast cancer cells [23]. In the current study, we focused on miR-20b-5p instead.

### Bioinformatics analysis of target genes

**Fig. 2:**
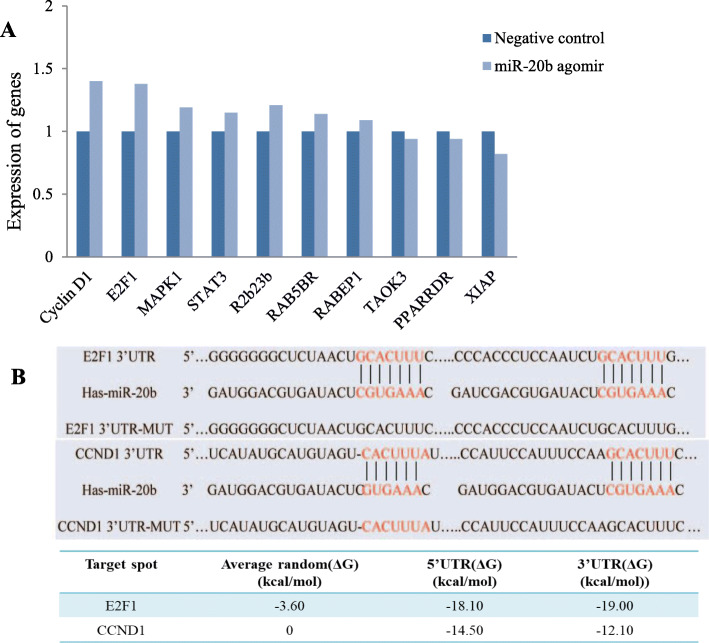
Bioinformatics analysis of target genes for miR-20b-5p. **a** qRT-PCR screening of potential target genes; **b** The binding site of miR-20b-5p to the E2F1–3′ UTR and CCND1–3′ UTR and free energy analysis

By bioinformatics analysis, we explored the target genes of miR-20b-5p related to cell proliferation and cell cycle and found 10 potential targets of miR-20b-5p, namely, CyclinD1 (CCND1), E2F1, MAPK1, STAT3, R2b23b, RAB5BR, RABEP1, TAOK3, PPARDR and XIAP. To roughly screen the potential target genes of miR-20b-5p, agomir-20b-5p was transfected into MCF-7 cells and the gene expression was detected by qRT-PCR. We found that miR-20b-5p increased the expression of some of the potential target genes while inhibited that of the others (Fig. 2a). The dramatically increased expression of CCND1 and E2F1 was intriguing and unexpected, since miRNAs usually negatively regulate their target genes. Therefore, the contradictory preliminary results of qRT-PCR compelled us to choose CCND1 and E2F1 for subsequent validation and functional studies. The binding free energy between CCND1 and miR-20b-5p at the 5′ UTR and 3′ UTR was − 14.50 kcal/mol and − 12.10 kcal/mol, respectively, lower than the average random free energy of CCND1 (0 kcal/mol). Likewise, the binding free energy between E2F1 and miR-20b-5p at the 5′ UTR and 3′ UTR was − 18.10 kcal/mol and − 19.00 kcal/mol, respectively, lower than the average random free energy of E2F1 (− 3.60 kcal/mol) (Fig. 2b) (Supplementary Fig. 2).

### Effects of miR-20b-5p on cell migration and wound healing ability

**Fig. 3:**
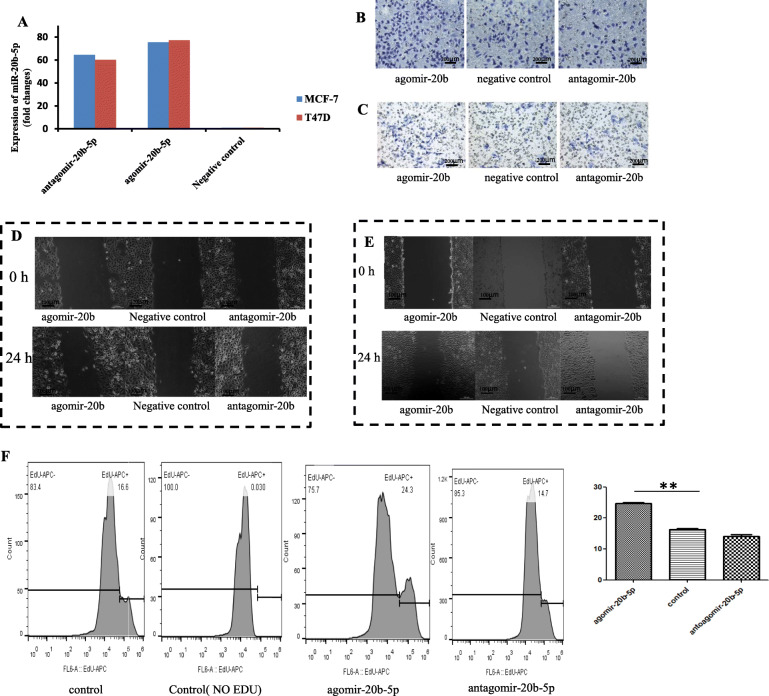
The effects of miR-20b-5p on migration in breast cancer cells. **a** Expression of miRNA after transient transfection; **b** Transwell migration experiment in MCF-7 cells; **c** Transwell migration experiment in T47D cells; **d** Wound healing assay in MCF-7 cells; **e** Wound healing assay in T47D cells; **f** Effects of miR-20b-5p on the proliferation of MCF-7 cells

To verify the regulatory effects of miR-20b-5p on migration and wound healing ability, miR-20b-5p was intervened from two sides in the current experiment. As expected, miR-20b-5p expression was increased 75-fold and 77-fold by agomir-20b-5p transfection into MCF-7 and T47D cells, respectively, and was downregulated 65-fold and 60-fold by transfection of antagomir-20b-5p into MCF-7 and T47D cells, respectively (Fig. 3a). Transwell migration experiments (*n* = 3) showed that agomir-20b-5p significantly promoted the migration ability of MCF-7 cells (85 ± 3 vs. 185 ± 2) (*P* < 0.01), and antagomir-20b-5p significantly inhibited the migration ability of MCF-7 cells (85 ± 3 vs. 62 ± 2) (*P* < 0.01) (Fig. 3b). Similarly, agomir-20b-5p significantly promoted the migration ability of T47D cells (20 ± 1 vs. 33 ± 2) (*P* < 0.01, *n* = 3), and antagomir-20b-5p markedly inhibited the migration ability of T47D cells (20 ± 1 vs. 14 ± 1) (*P* < 0.05, *n* = 3) (Fig. 3c).

For the wound healing ability assay, the ImageJ tool was used to measure the scratched surface, and the upper, middle and lower positions for the scratch were measured at 0 h and 24 h, respectively, and recorded as L_0_ and L_24_, based on which L = L_0_-L_24_ was calculated. The results showed that agomir-20b-5p significantly increased the wound healing ability of MCF-7 cells (161.6257 ± 11.23752 vs. 339.0160 ± 18.26367) (*P* < 0.01, *n* = 3), and antagomir-20b-5p inhibited the wound healing ability of MCF-7 cells (161.6257 ± 11.23752 vs. 120.0483 ± 16.72730), but the difference was not statistically significant (*P* > 0.05, *n* = 3) (Fig. 3d). Also, agomir-20b-5p significantly enhanced the wound healing ability of T47D cells (108.0030 ± 9.64574 vs. 435.9533 ± 37.53868) (*P* < 0.05, *n* = 3), while antagomir-20b-5p inhibited the wound healing ability of T47D cells but without a significant difference (108.0030 ± 9.64574 vs. 87.0583 ± 9.04403) (*P >* 0.05, *n* = 3) (Fig. 3e).

### Effects of miR-20b-5p on proliferation and apoptosis of BCSCs

The regulatory effects of miR-20b-5p on the proliferation of breast cancer cells was verified. In MCF-7 cells, EdU proliferation experiments indicated that agomir-20b-5p promoted cell proliferation, while antagomir-20b-5p inhibited cell proliferation, with a proliferation rate of 16.6% ± 0.68% in the control group, 24.3% ± 0.59% in the agomir-20b-5p group (*P* < 0.05, *n* = 3) and 14.7% ± 0.88% in the antagomir-20b-5p group (Fig. 3f).

**Fig. 4:**
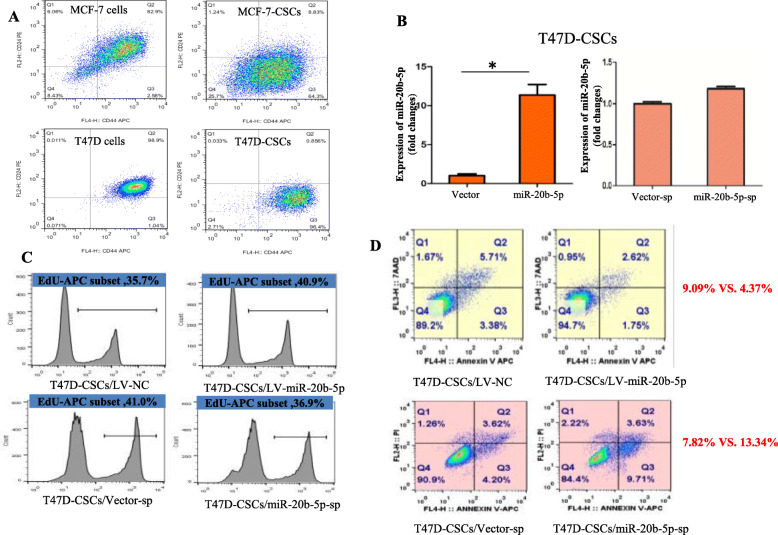
The effects of miR-20b-5p on proliferation and apoptosis in BCSCs. **a** The proportion of CD44^+^CD24^−/low^ subpopulation in breast cancer cells and BCSCs induced from MCF-7 and T47D cells; **b** Comparison of the expression of miR-20b-5p between lentivirus vector and miR-20b-5p and lentivirus-sp and miR-20b-5p-sp cells; **c** Effects of miR-20b-5p on the proliferation of T47D-CSCs; **d** Effects of miR-20b-5p on apoptosis of T47D-CSCs (Q2 + Q3)

The regulatory effects of miR-20b-5p on the proliferation of BCSCs was also investigated. The proliferation rate of the CD44^+^CD24^−/low^ subpopulation was 1.04 and 96.4% in T47D cells and induced T47D-CSCs, respectively (Fig. 4a). qRT-PCR analysis showed that miR-20b-5p expression was 11.20 times higher in T47D-CSCs/miR-20b-5p compared with T47D-CSCs/LV-NC, while no significant difference was observed between T47D-CSCs/miR-20b-5p-sp and T47D-CSCs/vector-sp (Fig. 4b). The EdU proliferation experiment in T47D-CSCs showed that the proliferation rate was 36.47% ± 1.87 and 41.63% ± 0.64% in the T47D-CSCs/LV-NC group and T47D-CSCs/LV-miR-20b-5p group (the proportion of cells in the S phase of the cell cycle), respectively (*P* = 0.011, *n* = 3), and was 41.80% ± 1.14 and 37.87% ± 0.95% in the T47D-CSCs/vector-sp group and T47D-CSCs/miR-20b-5p-sp group, respectively (*P* = 0.010, *n* = 3), indicating that miR-20b-5p increased the proliferation of T47D-CSCs (Fig. 4c).

Furthermore, the effect of miR-20b-5p on apoptosis of BCSCs was detected. The apoptosis rate was 8.56% ± 0.48 and 4.37% ± 0.45% in the T47D-CSCs/LV-NC group and T47D-CSCs/LV-miR-20b-5p group, respectively (*P* = 0.004, *n* = 3), while it was 7.43% ± 0.75 and 12.90% ± 0.54% in the T47D-CSCs/vector-sp group and T47D-CSCs/miR-20b-5p-sp group, respectively (*P* = 0.001, *n* = 3), indicating inhibition of apoptosis by miR-20b-5p in T47D-CSCs (Fig. 4d).

### Effects of miR-20b-5p on xenograft growth

**Fig. 5:**
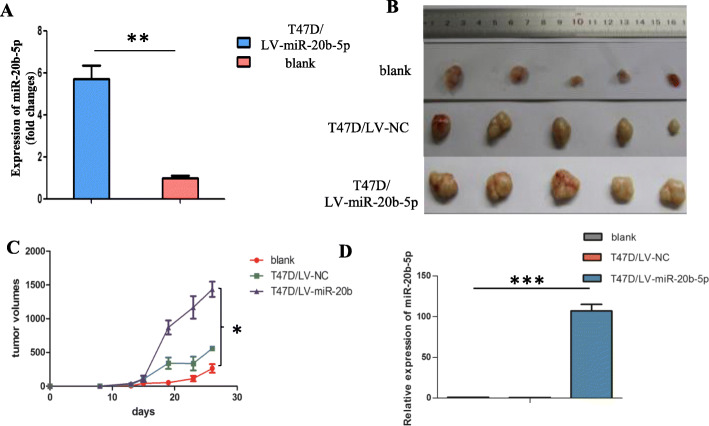
Xenograft growth promoted by miR-20b-5p upregulation. **a** Expression of miR-20b-5p after lentivirus transfection (The miR-20b-5p overexpression vector was labeled T47D-CSCs/LV-miR-20b-5p, and the control vector labeled T47D-CSCs/LV-NC); **b** Effects of miR-20b-5p on the tumorigenic ability of T47D cells; **c** Tumor growth curve (*P* < 0.05, *n* = 3); **d** qRT-PCR verified the expression of miR-20b-5p in xenografts (*P* < 0.001, *n* = 3)

The influence of miR-20b-5p on tumor growth was examined in vivo as well. After lentiviral transfection, miR-20b-5p expression was 5.618 times higher (Fig. 5a). The xenograft tumor volume in the miR-20b-5p overexpression group (T47D/LV-miR-20b-5p group) was significantly larger than that in the blank group and negative control group (T47D/LV-NC group) (Fig. 5b), and the growth curve consistently showed that the tumors in the T47D/LV-miR-20b-5p group grew significantly faster than those in the blank group and T47D/LV-NC group (*P* < 0.05, *n* = 3) (Fig. 5c). The expression of miR-20b-5p in xenografts in the T47D/LV-miR-20b-5p group was 107.68 times higher than that in the T47D/LV-NC group (*P* < 0.001, *n* = 3) (Fig. 5d).

### Verification of target genes

A crucial step to clarifying the regulatory mechanism of miR-20b-5p was to determine its target genes. Three experiments were conducted as follows.

**Fig. 6:**
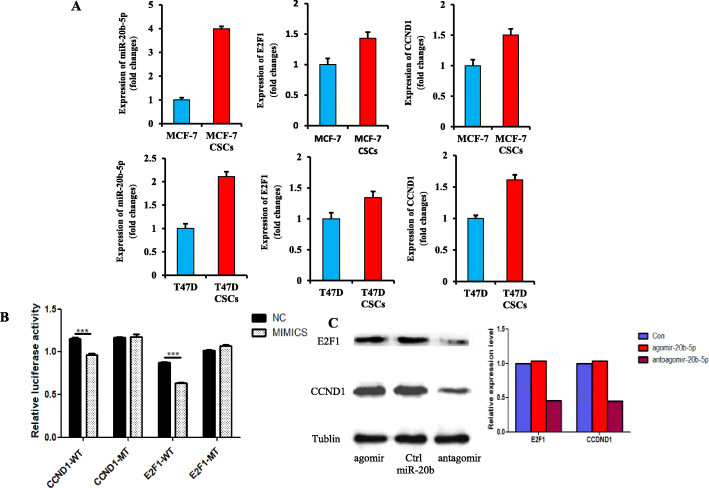
Verification of target genes of miR-20b-5p. **a** Comparison of the expression of miR-20b-5p, E2F1 and CCND1 between MCF-7 and MCF-7-CSCs, T47D and T47D-CSCs; **b** Dual luciferase assay to assess the regulatory effect of miR-20b-5p on CCND1 and E2F1; **c** CCND1 and E2F1 were regulated by miR-20b-5p overexpressed in MCF-7 cells, as demonstrated by western blot. (Full-length blots/gels are presented in Supplementary Figure 5)

First, we compared the correlation of miR-20b-5p expression with CCND1 and E2F1 mRNA levels between BCSCs and MCF-7 cells or T47D cells. The CD44^+^CD24^−/low^ subpopulation accounted for 2.58% of MCF-7 cells and 64.3% of induced MCF-7-CSCs (Fig. 4a). The expression of miR-20b-5p was 3.98 times and 2.21 times higher in BCSCs than in the control MCF-7 and T47D cells, respectively. Consistently, the mRNA expression levels of E2F1 and CCND1 were higher in BCSCs than in MCF-7 and T47D cells, although the difference was not statistically significant (Fig. 6a), thus confirming the regulatory relationship between miR-20b-5p and the two potential targets CCND1 and E2F1.

Second, the potential targeting of CCND1- and E2F1-WT was further determined by dual luciferase assay. Luciferase activity was significantly decreased in cells cotransfected with miR-20b-5p mimics plus CCND1-WT compared with cells cotransfected with NC plus CCND1-WT (0.966 ± 0.027 vs. 1.156 ± 0.026, *P* < 0.0001, *n* = 5). No significant difference was found between NC plus CCND1-MT and mimics plus CCND1-MT (1.164 ± 0.015 vs. 1.177 ± 0.051, *P* > 0.05, *n* = 5) (Fig. 6b). Likewise, the luciferase activity was significantly decreased in cells cotransfected with miR-20b-5p mimics plus E2F1-WT compared with cells cotransfected with NC plus E2F1-WT (0.636 ± 0.020 vs. 0.874 ± 0.015, *P* < 0.0001, *n* = 5). There was no significant difference between NC plus E2F1-MT and mimics plus E2F1-MT (1.015 ± 0.026 vs. 1.073 ± 0.018, *P* > 0.05, *n* = 5) (Fig. 6b). These results indicated that miR-20b-5p negatively regulated CCND1 and E2F1, validating the relationship between the miRNA and its direct targets.

Third, the protein expression levels of CCND1 and E2F1 were detected via western blot assay after agomir-20b-5p or antagomir-20b-5p transfection into MCF-7 cells. When the relative expression level of the control group was defined as 1, the relative expression levels of E2F1 and CCND1 were both 1.04 in the agomir-20b-5p group. The relative expression level of CCND1 and E2F1 in the antagomir-20b-5p group decreased to 0.45 and 0.46, respectively (Fig. 6c). This western blot assay demonstrated that miR-20b-5p increased the protein expression of CCND1 and E2F1.

Based on all the three results above, it seemed that miR-20b-5p directly downregulated the targets of CCND1 and E2F1, but indirectly upregulated these two proteins.

## Discussion

We made researches on the reports on the miR-20b family in different tumors. The expression level of miR-20b was reported to be increased in gastric cancer [16], breast cancer [17, 18] and hepatocellular carcinoma [19]. Wang B et al. [25] found that miR-20b promoted proliferation, migration, invasion and tumorigenesis in esophageal cancer cells. It was also reported that the aberrant expression of miR-20b contributed to tumorigenesis and progression of breast cancer [26]. In the above literatures, miR-20b is shown to play an oncogenic role. However, miR-20b includes both miR-20b-3p and miR-20b-5p, which might have different biological functions. Only one report showed that overexpressed miR-20b-5p in cancer tissue and patient serum had a regulatory effect on the proliferation and migration of breast cancer cells [27]. In addition, there has been no exploration of the regulatory role of miR-20b in BCSCs. In the current study, we focused on the function of miR-20b-5p and found that miR-20b-5p acted as an oncogene in breast cancer and malignant BCSCs.

Cancer stem cells (CSCs) are capable of self-renewal and multidirectional differentiation [28]. Studies have shown that the molecular regulation of BCSCs is different from that of breast cancer cells [29]. BCSCs are associated with epithelial-mesenchymal transformation (EMT), miRNAs, the tumor microenvironment and other factors [30]. Our experimental results indicated that the upregulation of miR-20b-5p in MCF-7 and T47D cells significantly promoted the migration ability of breast cancer cells. Furthermore, overexpression and knockdown systems of miR-20b-5p in BCSCs were established. It was confirmed that miR-20b-5p promoted the proliferation of BCSCs and inhibited the apoptosis of BCSCs. As an integrated outcome of miR-20b-5p on breast cancer cells and stem cells, the xenografts of breast cancer cells were significantly enlarged by miR-20b-5p overexpression, which indicated that miR-20b-5p acted as an oncogene in vivo. Similarly, one study has demonstrated that miR-128-3p inhibits the stem-like cell features of BCSCs via inhibition of the Wnt signaling pathway by downregulating NEK2, which provides a new target for breast cancer treatment [31]. Then, another study indicates that CD24 and CD44 are cancer stem cells which can promote the development of breast cancer [32]. Many miRNAs have been shown to regulate the self-renewal and differentiation of CSCs, such as the let-7 miRNA family [33, 34]. The let-7 miRNA family appears to play a substantial role in the CSC phenotype. In breast cancer, let-7 is found to be downregulated. In normal tissues, it regulates self-renewal, acting as a pro-differentiation miRNA, whereas in breast cancer it is repressed by the Wnt/β-catenin pathway. Therefore, we also compared its expression difference between normal tissues and tumor tissues (http://mirnamap.mbc.nctu.edu.tw/) in our study, and found that the expression of miR-20b-5p in normal tissues was also different. The expression level was high in the prostate, kidney, lung and other normal tissues, and was low in the liver, adipose, muscle. The highest expression level was in the thymus. No expression was observed in breast. (Supplementary Fig. 3).

Although several researches showed that miR-20b-5p acts as an oncogenic molecule in various tumors, we successfully screened BCSCs-associated miRNA profiles and identified miR-20b-5p as a key molecule in mediating BCSCs function. To our best knowledge, it’s the first time that miR-20b-5p has been proved to have important effects on BCSCs. It’s a novel regulation mechanism for the malignant progression of BCSCs, such as proliferation and apoptosis.

To further clarify the underlying mechanism of miR-20b-5p in the regulation of breast cancer cells and stem cells, we chose CCND1 and E2F1 as potential targets based on bioinformatics analysis and preliminary screening by qRT-PCR. CCND1, as an oncogene, is overexpressed in tumors and plays an important regulatory role in normal breast development, damage repair, maintenance of breast epithelial stem cell proliferation and self-renewal [35–37]. E2F1, a member of the E2F transcription factor family, regulates gene expression related to cell proliferation, differentiation and apoptosis and controls the cell cycle via a two-way regulatory mechanism. As a tumor suppressor gene or oncogene, E2F1 is closely related to tumor progression and drug resistance [38–41]. The literature shows that CCND1 and E2F1 are regulated by the miR-17-92 cluster, which is a miRNA cluster located on chromosome 13 that is composed of miR-17, miR-20a and miR-92a-1 [8, 9]. This suggests that E2F1 is targeted by certain miRNAs and is involved in the regulation of tumor cells [42]. To confirm that miR-20b-5p regulated CCND1 and E2F1 expression, we performed three experiments, namely, a comparison between BCSCs and the control breast cancer cells, a dual-luciferase assay and western blot analysis after overexpression or knockdown of miR-20b-5p. The dual-luciferase reporting system showed that CCND1 and E2F1 were the real targets of miR-20b-5p. Our western blot analysis showed that antagomir-20b-5p significantly decreased the protein levels of both CCND1 and E2F1. The luciferase activity was inhibited by miRNA mimics, indicating that miR-20b-5p downregulated the targets. On the contrary, if the luciferase activity was enhanced by miRNA mimics, it may suggest that miR-20b-5p upregulated the targets. Additionally, western blot assay may not demonstrate a direct regulation, as it is a comprehensive reflection of direct and indirect regulation by the miRNA.

This seems to be a contradictory but interesting phenomenon. One speculation is that the observed upregulation of CCND1 and E2F1 might well be the side effect of the upregulation of miR-20b-5p. Theoretically a high level of miR-20b-5p may cause the occupation of the RNA-induced silencing complex (RISC) by this miRNA and thus RISCs are less available for other miRNAs. Namely, the mature miRNA (the guide strand) is incorporated into RISCs, whereas the passage miRNA* strand can be loaded in the RISC as well or degraded [43]. The mature miRNA guides the Argonaute protein of the RISC to the complementary mRNA sequence on the target to repress its expression [44]. In this case, some genes targeted by other miRNAs might get released from their negative regulation and might get upregulated [45]. Another speculation is that the antagomirs or mimic will produce off-target effects [46]. Off-target phenomenon usually occurs when miR is overexpressed more than hundreds of folds. In our experiment, we used agomir to make miR overexpression less than 80 folds, which is a proper range and effectively avoids off-target effects. Therefore, the second speculation is out of our consideration.

Our results revealed an indirect upregulation of CCND1 and E2F1 by miR-20b-5p. There are likely to be unknown targets (X and Y) negatively regulated by miR-20b-5p. The unknown targets (X and Y) could inhibit CCND1 and E2F1 protein expression. Overall, miR-20b-5p regulated both CCND1 and E2F1 via bidirectional regulation, namely, direct downregulation and indirect upregulation. As a tug-of-war mechanism, the indirect promoting effect of miR-20b-5p on CCND1 and E2F1 may outweigh its direct downregulation of CCND1 and E2F1. However, it is still necessary to reveal the underlying mechanism, which will be explored in further studies. The hypothesis involving the effect of miR-20b-5p on CCND1 and E2F1 is shown in a regulatory network (Supplementary Fig. 4).

## Conclusion

In summary, this study shows that miR-20b-5p can promote tumorigenesis in both breast cancer cells and stem cells. We speculate that the underlying mechanism of miR-20b-5p contributing to the malignant progression of breast cancer is that miR-20b-5p overall upregulates both CCND1 and E2F1 via bidirectional regulation.

## Supplementary information

**Additional file 1: Supplementary Figure S1.** A: The sequences of CCND1 (19068-1) CCND1 NM_053056-3utr(mir-20b)-3mu GV306 and CCND1 (24297-2) CCND1 NM_053056-3utr(mir-20b)-3 GV306; B: The sequences of E2F1 (19067-1) E2F1 NM_005225-3utr(mir-20b)-3mu GV306 and E2F1 (19070-2) E2F1 NM_005225-3utr(mir-20b)-3 GV306. **Supplementary Figure S2.** The predicted binding site and binding free energy between miR-20b-5p and the targets CCND1 (A) and E2F1 (B). **Supplementary Figure S3.** The expression of miR-20b in normal tissues. **Supplementary Figure S4.** The putative regulatory mechanism involving miR-20b-5p and two target genes, CCND1 and E2F1. X is the intermediate molecule of E2F1 indirectly regulated by miR-20b-5p, and Y is the intermediate molecule of CCND1 indirectly regulated by miR-20b-5p. **Supplementary Figure S5.** Uncropped full-length gels and western blot assay.

## Data Availability

All data generated or analyzed during this study are included in this article.

## Abbreviations

miRNAs: MicroRNA
BCSCs: Breast cancer stem cells
FBS: Fetal bovine serum
GO: Gene Ontology
CCND1: Cyclin D1
E2F1: E2F transcription factor 1
Edu: 5-ethynyl-2′-deoxyuridine
T47D-CSCs: T47D cancer stem cells
sp: Sponge
LV: Lentivirus
NC: Negative control
