# Supplementary material for: Oncogenic miR-20b-5p contributes to malignant behaviors of breast cancer stem cells by bidirectionally regulating CCND1 and E2F1
Source: BMC Cancer. 2020 Oct 2;20:949. doi: 10.1186/s12885-020-07395-y (PMC7531112; doi:10.1186/s12885-020-07395-y)
Supplement: Supplementary file 1 — Additional file 1: Supplementary Figure S1. A: The sequences of CCND1 (19068-1) CCND1 NM_053056-3utr(mir-20b)-3mu GV306 and CCND1 (24297-2) CCND1 NM_053056-3utr(mir-20b)-3 GV306; B: The sequences of E2F1 (19067-1) E2F1 NM_005225-3utr(mir-20b)-3mu GV306 and E2F1 (19070-2) E2F1 NM_005225-3utr(mir-20b)-3 GV306. Supplementary Figure S2. The predicted binding site and binding free energy between miR-20b-5p and the targets CCND1 (A) and E2F1 (B). Supplementary Figure S3. The expression of miR-20b in normal tissues. Supplementary Figure S4. The putative regulatory mechanism involving miR-20b-5p and two target genes, CCND1 and E2F1. X is the intermediate molecule of E2F1 indirectly regulated by miR-20b-5p, and Y is the intermediate molecule of CCND1 indirectly regulated by miR-20b-5p. Supplementary Figure S5. Uncropped full-length gels and western blot assay. [file 12885_2020_7395_MOESM1_ESM.pdf]

CCND1-WT

NM_053056-3utr(mir-20b)-3

TTTTAGGTAGAAAAACATATTCTTGTGCTTTTCCTGATAAAGCACAGCTGTAGTGGGGTTCTAGGCA

TCTCTGTACTTTGCTTGCTCATATGCATGTAGTCACTTTATAAGTCATTGTATGTTATTATATTCCGTAG

GTAGATGTGTAACCTCTTCACCTTATTCATGGCTGAAGTCACCTCTTGGTTACAGTAGCGTAGCGTG

CCTTTTCTACCCAACGGCCCTGCAGCCAGCTCACGTCCAGGTTCAACCCACAGCTACTTGGTTTGT

GTTCTTCTTCATATTCTAAAACCATTCCATTTCCAAGCACTTTCAGTCCAATAGGTGTAGGAAATAGC

GCTGTTTTTGTTGTGTGTGCAGGGAGGGCAGTTTTCTAATGGAATGGTTTGGGAATATCCATGTACT

TGTTTGCA

CCND1-MT

NM_053056-3utr(mir-20b)-3mu

TTTTAGGTAGAAAAACATATTCTTGTGCTTTTCCTGATAAAGCACAGCTGTAGTGGGGTTCTA

GGCATCTCTGTACTTTGCTTGCTCATATGCATGTAGTATAAGTCATTGTATGTTATTATATTCCGT

AGGTAGATGTGTAACCTCTTCACCTTATTCATGGCTGAAGTCACCTCTTGGTTACAGTAGCGT

AGCGTGCCTTTTCTACCCAACGGCCCTGCAGCCAGCTCACGTCCAGGTTCAACCCACAGCTA

CTTGGTTTGTGTTCTTCTTCATATTCTAAAACCATTCCATTTCCAACAGTCCAATAGGTGTAGG

AAATAGCGCTGTTTTTGTTGTGTGTGCAGGGAGGGCAGTTTTCTAATGGAATGGTTTGGGAAT

ATCCATGTACTTGTTTGCA

E2F1-WT

NM_005225-3utr(mir-20b)-3

GAAGGTGAACATACACCTCTGTGTGTGCACTGCAGACACGCCCCAGTGTGTCCACATGTGTG

TGCATGAGTCCATGTGTGCGCGTGGGGGGGCTCTAACTGCACTTTCGGCCCTTTTGCTCTGGG

GGTCCCACAAGGCCCAGGGCAGTGCCTGCTCCCAGAATCTGGTGCTCTGACCAGGCCAGGTG

GGGAGGCTTTGGCTGGCTGGAGGAGGGGTGGTGAAGGCCTCCCCCAGCCCAGACCCTGTGG

TCCCTCCTGCAGTGTCTGAAGCGCCTGCCTCCCCACTGCTCTGCCCCACCCTCCAATCTGCAC

TTTGATTTGCTTCCTAACAGCTCTGTTCCCTCCTGCTTTGGTTTTAATAAATATTTTGATGACGT

TTGGGCCGGGTTTTGGGACTCTGTTGGGAACATTTCGG

E2F1-MT

NM_005225-3utr(mir-20b)-3mu

GAAGGTGAACATACACCTCTGTGTGTGCACTGCAGACACGCCCCAGTGTGTCCACATGTGTGTG

CATGAGTCCATGTGTGCGCGTGGGGGGGCTCTAACTCGGCCCTTTTGCTCTGGGGGTCCCACAAG

GCCCAGGGCAGTGCCTGCTCCCAGAATCTGGTGCTCTGACCAGGCCAGGTGGGGAGGCTTTGGC

TGGCTGGAGGAGGGGTGGTGAAGGCCTCCCCCAGCCCAGACCCTGTGGTCCCTCCTGCAGTGTC

TGAAGCGCCTGCCTCCCCACTGCTCTGCCCCACCCTCCAATCTGATTTGCTTCCTAACAGCTCTGT

TCCCTCCTGCTTTGGTTTTAATAAATATTTTGATGACGTTTGGGCCGGGTTTTGGGACTCTGTTGG

GAACATTTCGG

Supplementary figure 1: A: The sequences of CCND1(19068-1) CCND1 NM_053056-3utr(mir-20b)-3mu GV306 and CCND1(24297-2) CCND1 NM_053056-3utr(mir-20b)-3 GV306; B: The sequences of E2F1(19067-1) E2F1 NM_005225-3utr(mir-20b)-3mu GV306 and E2F1(19070-2) E2F1 NM_005225-3utr(mir-20b)-3 GV306

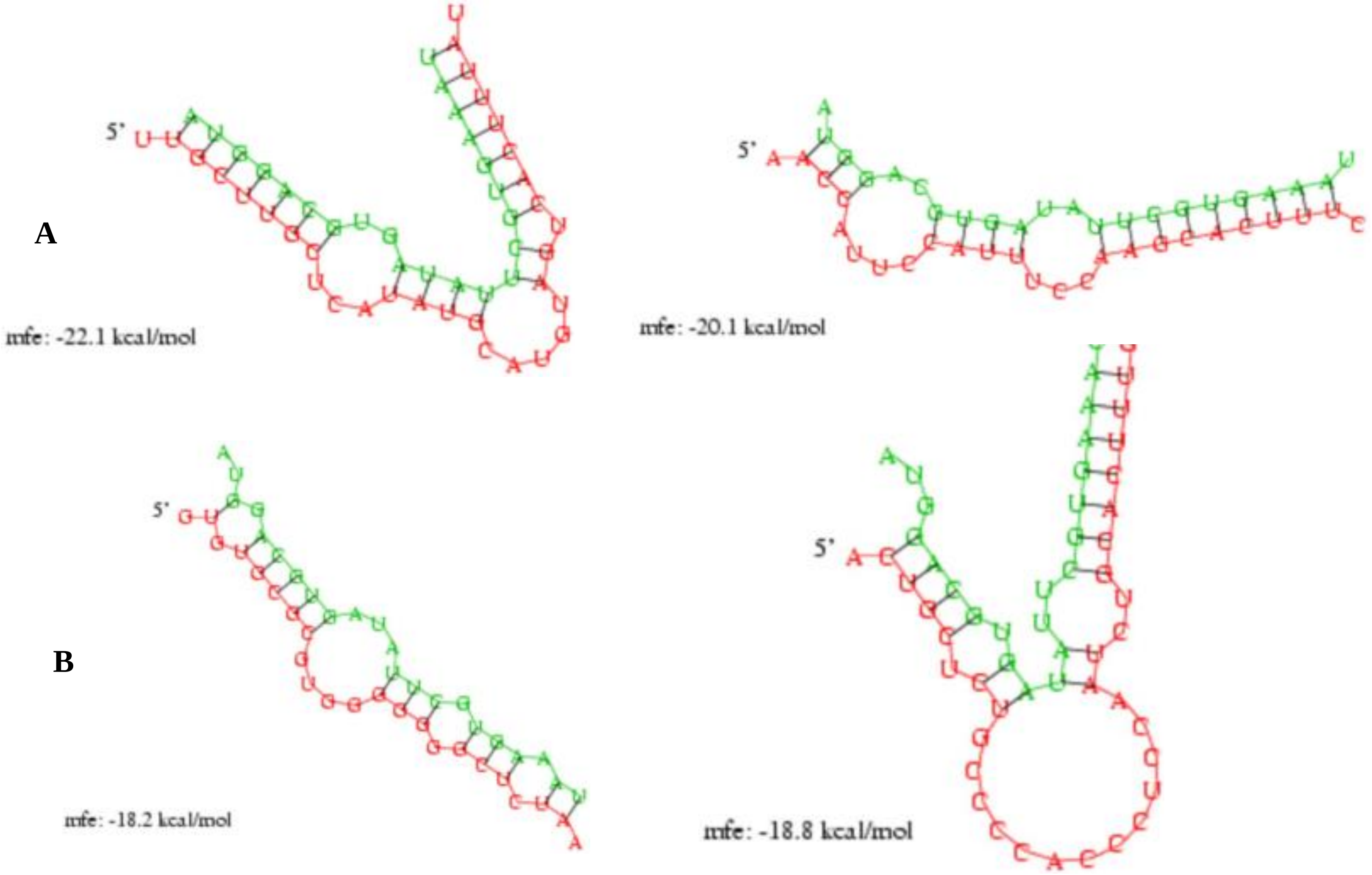
A
5'
mfe: -22.1 kcal/mol
5'
mfe: -20.1 kcal/mol
B
5'
mfe: -18.2 kcal/mol
5'
mfe: -18.8 kcal/mol

Supplementary figure 2: The predicted binding site and binding free energy between miR-20b-5p and the targets CCND1 (A) and E2F1 (B).

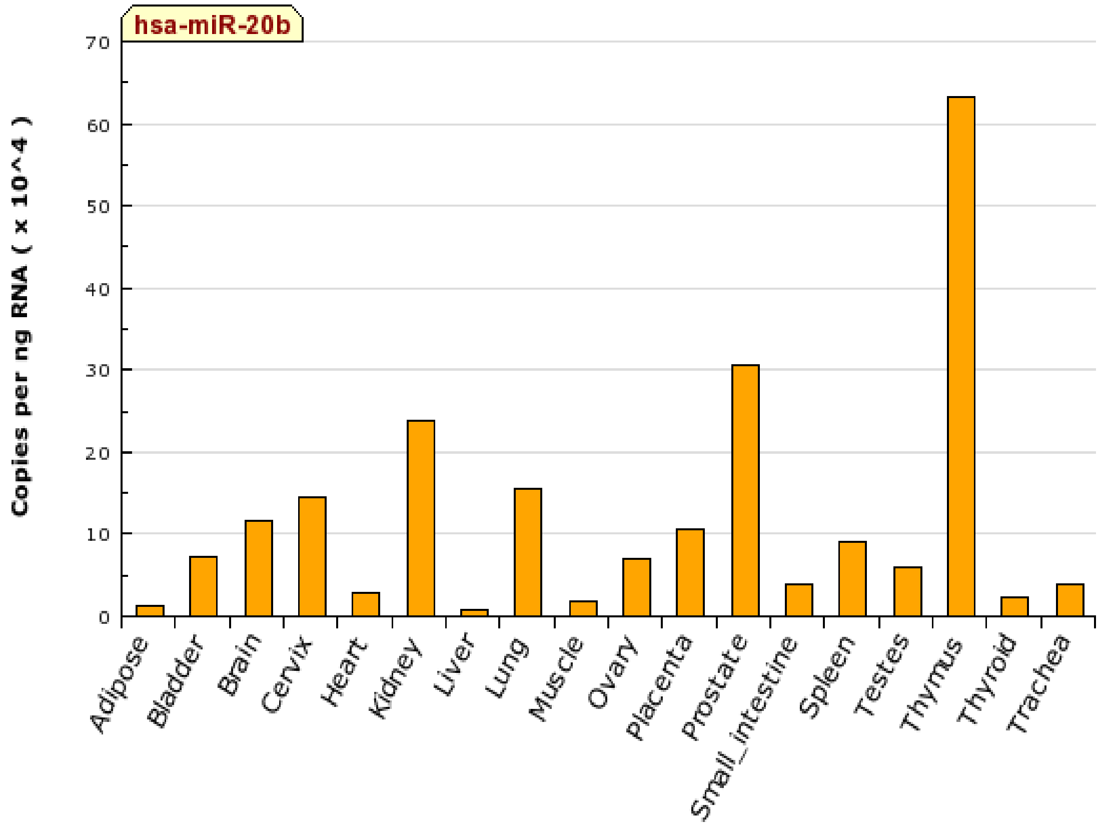

Supplementary figure 3: The expression of miR-20b in normal tissues.

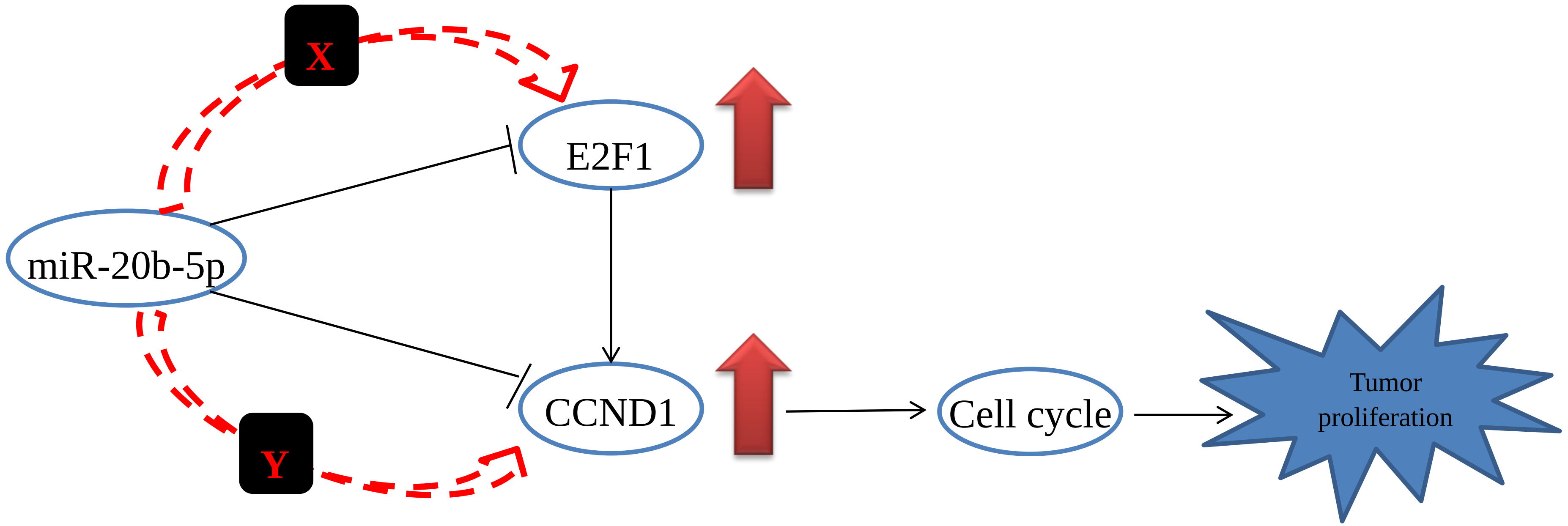

Supplementary figure 4: The putative regulatory mechanism between miR-20b-5p and two target genes CCND1 and E2F1, X is the intermediate molecule of E2F1 indirectly regulated by miR-20b-5p, Y is the intermediate molecule of CCND1 indirectly regulated by miR-20b-5p.

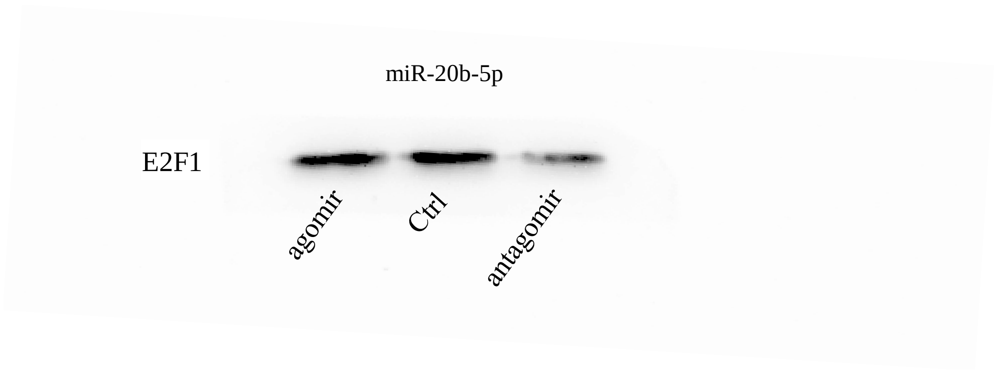

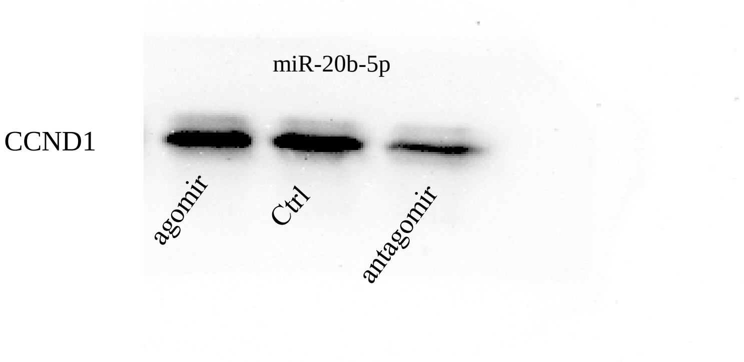

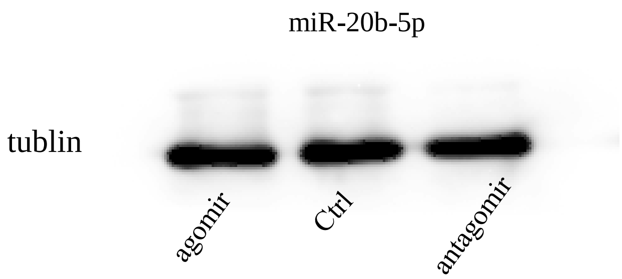

Supplementary figure 5: Uncropped full-length gels and western blot assay.
